# Functional Engineering of Bioactive Peptides: Chemical Modifications and Synthetic Biology Approaches

**DOI:** 10.3390/ijms27135939

**Published:** 2026-07-01

**Authors:** Liangjie Hu, Zhimin Zhang, Xinxi Li, Yisheng Liang, Ruibo Huang, Li Wen

**Affiliations:** School of Food Science and Bioengineering, Hunan Provincial Key Laboratory of Cytochemistry, Changsha University of Science & Technology, Changsha 410114, China

**Keywords:** bioactive peptides, chemical modification, synthetic biology modification, physiological activity

## Abstract

Bioactive peptides (BPs) are widely distributed and exhibit remarkable physiological activities. However, their natural forms are frequently characterized by short half-lives, low membrane permeability, poor stability, and inadequate oral bioavailability, which severely limit their applications in the food, pharmaceutical, and biomaterial fields. Therefore, modification and engineering of natural BPs are essential to surmount these inherent limitations. Synthetic biology-based modification strategies, including amino acid substitution, sequence truncation and hybridization, side-chain functionalization, and main-chain/side-chain integration, are comprehensively summarized in this review. Chemical modification strategies, such as terminal modification, cyclization, backbone modification, polymer conjugation, lipidation, and glycosylation, are also discussed, with particular attention to their advantages, potential drawbacks, and practical limitations. Based on 122 studies identified through systematic literature searches across major scientific databases, this review also discusses the current challenges and future trends in BP modification, providing theoretical guidance and innovative insights for the further development and enhanced utilization of BPs.

## 1. Introduction

Bioactive peptides (BPs) are a class of small-molecule protein fragments with specific physiological functions, typically composed of 2–20 amino acid residues [1]. However, under specific conditions, longer peptide chains may occur, such as the soybean-derived peptide lunasin, which consists of 43 amino acid residues [2]. BPs are derived from diverse sources, including natural extraction, proteolytic digestion, and chemical synthesis [3,4]. They exhibit antimicrobial [5], antioxidant [6], antihypertensive [7] immunomodulatory [8], and skin-repairing activities [9]. Although antimicrobial peptides are frequently discussed in this review because they represent a well-studied model system for peptide engineering, the scope of this review is not limited to antimicrobial peptides. Instead, this review focuses on structural modification strategies that are broadly applicable to diverse BP classes, including antimicrobial, antihypertensive, antioxidant, immunomodulatory, endocrine/metabolic, and other functional peptides. Research indicates that BP functions primarily depend on amino acid composition, sequence framework, and secondary structure [10,11]. For instance, cationic peptides rich in basic amino acids disrupt bacterial membranes to exert antibacterial activity [12,13]; peptides containing aromatic residues exhibit strong free radical scavenging capacity [14,15]. The establishment of databases and screening platforms such as BIOPEP-UWM and FeptideDB has accelerated the identification and structural optimization of BPs [16,17,18]. Although natural BPs often exhibit significant activity, their in vivo application remains constrained by issues such as susceptibility to enzymatic degradation, short half-lives, limited membrane permeability, low oral bioavailability, and potential immunogenicity. These limitations are mainly caused by the amide bonds in peptide structures, which are easily broken, as well as by their small size and high attraction for water. As a result, they are either swiftly eliminated from the body or find it difficult to cross biological barriers in vivo [19,20,21]. However, the food and pharmaceutical industries continue to use BPs as natural functional ingredients or medication candidates in product development [22,23].

The structural optimization techniques, including PEGylation (polyethylene glycol modification), cyclization, fatty acid substitution and D-amino acid replacement, that have been widely developed in recent years to improve the application and production of BPs are summarized in this review. Focus is placed on clarifying how they improve pharmacokinetics, increase stability and extend residence duration in vivo. Potential development paths are described, and how well the intrinsic constraints of native bioactive peptides are overcome by these modification techniques is thoroughly assessed. It is anticipated that this study will broaden the perspectives for further exploration of the physiological activities of bioactive peptides and their prospective applications in various fields.

This review was conducted based on a systematic literature search of major scientific databases, including PubMed, Web of Science, and Science Direct. The literature was primarily focused on studies published within the last 10 years, with greater emphasis on the most recent 3 years to ensure the inclusion of up-to-date research developments. Relevant articles were selected according to their relevance to bioactive peptides and peptide structural modification, and studies that did not meet the scope or scientific relevance of this review were excluded.

## 2. Chemical Modification Strategies for Bioactive Peptides

### 2.1. Main Chain Modification

#### 2.1.1. Terminal Modification

A key tactic for improving the pharmacokinetic properties and in vivo stability of peptide molecules is main chain modification, of which terminal protection is one of the most traditional and often used techniques. Common terminal modifications include N-terminal acetylation, PEGylation and C-terminal amidation. These modifications shield peptide termini from degradation by exopeptidases and aminopeptidases [24], thereby enhancing stability. The antimicrobial peptide L163 is susceptible to degradation in plasma, but N-terminal acetylation enhances its tolerance to varying pH levels, plasma conditions and trypsin degradation [25]. Furthermore, the 20-residue linear peptide A20FMDV2 has been demonstrated as a potential therapeutic drug carrier. Introducing d-aspartic acid at its N-terminus and d-threonine at its C-terminus into the A20FMDV2 variant further enhances biological activity and significantly reduces its susceptibility to plasma degradation [26].

However, these two modifications have certain limitations. N-terminal acetylation may weaken the electrostatic interactions between the peptide and the cell membrane or receptors, thereby reducing cell penetration and decreasing certain biological activities. Moreover, certain acetylated peptides may be recognized by the Ac/N-end rule pathway, leading to accelerated degradation. Conversely, C-terminal amidation can enhance α-helix stability and membrane-binding ability. Nevertheless, it may also increase hydrophobicity and cytotoxicity or hemolytic activity. It may further complicate peptide synthesis and purification. Furthermore, both modifications block terminal active sites, thereby limiting subsequent functionalizations such as PEGylation, fluorescent labeling, or fatty acid conjugation [20,27,28].

**Figure 1:**
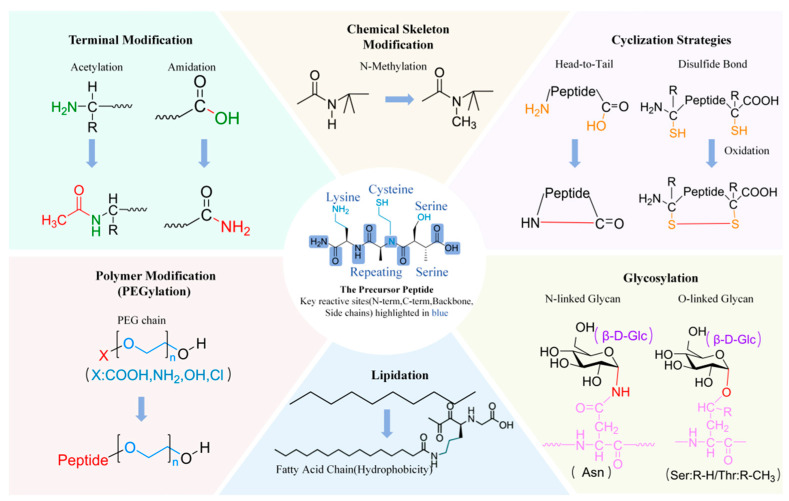
Schematic illustration of chemical modification strategies of bioactive peptides.

The outline of bioactive peptide modification options is illustrated in Figure 1 including side-chain modification, main-chain modification, and synergistic optimization of the main and side chains.

#### 2.1.2. Cyclization Strategies

As another prevalent peptide engineering tactic, cyclization functions to boost peptide resistance against enzymatic degradation and reinforce structural integrity through confinement of the polypeptide chain’s conformational flexibility. Studies show that head-to-tail cyclization significantly increases peptide stiffness, reduces conformational flexibility, enhances affinity for target receptors, and extends in vivo stability [29]. Methodologically, late-stage cyclization techniques catalyzed by transition metals have recently attracted substantial interest. Among these methods, effective peptide macrocyclization is achievable through Pd(II)-catalyzed C-H activation, generating cyclic peptides with pseudopeptide properties. In anticancer peptide studies, these modifications have demonstrated several-fold enhancements in biological activity [30]. Functionally, cyclization stabilizes the α-helix or β-sheet structures of naturally occurring cyclic peptides, including cyclic oligopeptides, and increases their membrane permeability and receptor-binding capabilities [31]. In addition, the aforementioned approach lends itself to rational structural modification of antimicrobial peptides. Cumulative research indicates that cyclization is capable of extending peptides’ tolerance toward proteolytic degradation from a minute-scale lifespan to several hours, thereby markedly elevating structural stability and in vivo application prospect [32]. Overall, cyclization improves structural stability, target-binding affinity, membrane permeability, and half-life. It is therefore considered one of the most effective strategies for enhancing the drugability of peptide drugs [33].

However, in practical applications, cyclization still faces several challenges, including insufficient cyclization efficiency, difficulties in precisely controlling ring size and conformation, limited stereoselectivity, and high costs associated with synthesizing complex macrocyclic structures [33]. Additionally, different cyclization methods can significantly influence peptide spatial conformation and biological activity. In certain cases, excessive conformational constraints may reduce the flexibility necessary for target binding, thus negatively impacting pharmacological activity [29]. Furthermore, as cyclic peptide structural complexity increases, challenges related to large-scale synthesis, purification, and quality control grow, further hindering the industrialization of certain cyclic peptide candidates. Despite the increased synthetic complexity, several cyclic peptide drugs, including cyclosporine, ziconotide, and octreotide, have successfully entered clinical practice. These examples demonstrate the clinical feasibility of cyclic peptide design.

Future developments in cyclization will therefore focus more heavily on establishing highly selective cyclization reactions, precise design of complex cyclic peptides, and integrated application of AI-assisted conformational optimization. The objective is to improve structural stability, biological activity, and developability in a coordinated manner. This would support the development and clinical translation of next-generation high-performance cyclic peptide drugs.

#### 2.1.3. Chemical Skeleton Modification

Backbone engineering involves extensive chemical alterations, such as scaffold replacement, in addition to terminal protection and cyclization modifications. These strategies aim to increase peptide metabolic stability and reduce protease recognition. Common approaches include replacing amide bonds within the peptide backbone with reduced amines, ether bonds, or other isosteric substitutes, thus altering bond polarity and flexibility and lowering susceptibility to hydrolysis [34,35]. Among these modifications, N-methylation mimics structural features of non-ribosomal peptides and decreases amide hydrogen bonding. This modification has been shown to extend the in vivo half-life of long-acting antidiabetic peptides and various therapeutic peptides [36]. Additionally, introducing non-natural bonds, such as fluorinated or selenated bonds, into the backbone enhances oxidative stability, offering distinct advantages in antioxidant peptide design [35]. Overall, the primary benefit of backbone replacement lies in its capacity to directly inactivate protease recognition sites at the molecular level, substantially improving proteolytic resistance, serum stability, and bioavailability. These modifications also enhance transmembrane transport and tissue distribution, thereby expanding the clinical application potential of peptide drugs.

Peptoid-based backbone engineering represents an important class of peptidomimetic strategies. Unlike conventional peptides, peptoids consist of N-substituted glycines, in which side chains are attached to the backbone nitrogen rather than the α-carbon. This structural alteration eliminates backbone hydrogen-bond donors and reduces recognition by proteolytic enzymes, thereby markedly enhancing metabolic stability and resistance to enzymatic degradation. In antimicrobial applications, peptoids have demonstrated broad-spectrum activity and antibiofilm effects by preserving the amphiphilic and cationic characteristics of natural antimicrobial peptides while overcoming their key limitations, such as rapid proteolysis. Furthermore, the tunable chemical diversity of N-substituted side chains allows precise modulation of hydrophobicity, charge distribution, membrane interactions, and cytotoxicity, making peptoids promising candidates for next-generation antimicrobial peptide mimetics [37,38,39].

However, this strategy has several limitations. First, excessive or inappropriate backbone modifications may disrupt the natural spatial conformation of peptides and critical receptor-binding sites, leading to reduced or complete loss of biological activity. Second, the introduction of non-natural bonds typically increases synthesis and purification complexity, raises overall production costs, and may lead to potential risks such as immunogenicity, altered metabolic pathways, and insufficient long-term safety evaluation [40]. Furthermore, due to significant variation in structural tolerance to backbone substitutions among different peptides, developing uniform, universally applicable design principles remain challenging. Consequently, fine-tuning usually requires integrating structure–activity relationship (SAR) analysis, molecular dynamics simulations, and systematic experimental validation [41,42].

Currently, these backbone engineering strategies have been applied to various bioactive peptides, including anticancer, antimicrobial, and antihypertensive peptides, showing significant enhancements in resistance to enzymatic degradation and drug development potential. However, achieving an optimal balance between structural stability and biological function remains a key direction for future research.

### 2.2. Modification of Side Chains and Functional Groups

#### 2.2.1. Macromolecular Polymer Modification

PEGylation is a representative macromolecular polymer modification strategy. Its primary mechanism involves the formation of a highly hydrated steric barrier on the molecular surface, which shields protease cleavage sites and improves pharmacokinetic properties. This approach has been successfully applied to various peptide and protein therapeutics. For example, PEG conjugation of interferon significantly extends its in vivo therapeutic effect [43]. Mechanistically, PEG chains are highly flexible and hydrophilic. In aqueous environments, they form a stable hydration layer. This layer increases the apparent molecular size of the drug, reduces glomerular filtration, and decreases uptake and clearance by the reticuloendothelial system. These effects prolong the circulation half-life. In addition, the steric hindrance provided by PEG effectively reduces recognition by proteases, antibodies, and immune cells, thereby enhancing in vivo stability and bioavailability. The major advantages of PEGylation include improved aqueous solubility, enhanced thermal [44] and storage stability [45], prolonged circulation time, and reduced dosing frequency [46]. Consequently, it has become one of the most widely used chemical modification strategies for the development of long-acting peptide therapeutics.

However, as research has advanced, several limitations have become evident. First, the introduction of PEG chains may generate steric hindrance around active sites or induce conformational changes in peptide molecules, thereby reducing biological activity. Second, accumulating evidence suggests that pre-existing or treatment-induced anti-PEG antibodies [47,48] can trigger the accelerated blood clearance (ABC) effect [49], resulting in reduced therapeutic efficacy and an increased risk of hypersensitivity reactions. Furthermore, long-term administration of high-molecular-weight PEG may lead to intracellular accumulation and vacuolization, raising concerns regarding long-term safety.

Therefore, although PEGylation remains one of the most mature polymer modification strategies, concerns related to immunogenicity, reduced bioactivity, and long-term safety have encouraged the development of novel non-PEG polymer systems with improved therapeutic performance and biocompatibility.

#### 2.2.2. Lipid-Based Modification

Lipidation is an important modification strategy that effectively improves the in vivo stability and pharmacokinetic performance of peptide drugs. It is typically achieved by covalently attaching C12-C20 fatty acid chains to peptide molecules. This modification enhances reversible binding to serum albumin, thereby reducing renal clearance and significantly prolonging circulation time [50]. This strategy has been successfully applied to several GLP-1 analogs, including Liraglutide and Semaglutide. In these drugs, lipid-mediated albumin binding is regarded as a key mechanism underlying their extended duration of action [51]. Mechanistically, lipidation increases peptide hydrophobicity, which not only promotes albumin binding but also strengthens interactions with cell membranes. As a result, cellular uptake efficiency and membrane insertion capacity are improved. In antimicrobial peptide design, this modification has been associated with enhanced membrane penetration and antimicrobial activity [52]. Furthermore, studies have shown that, when fatty acid chain length and linkage chemistry are appropriately controlled, lipidation combined with permeability-enhancing strategies may improve the oral bioavailability of certain peptide drugs. However, inappropriate design may lead to membrane toxicity or nonspecific aggregation [53].

Despite its advantages in prolonging drug action, lipid modification also has several limitations. The introduction of fatty acid chains markedly increases peptide hydrophobicity. Excessive lipidation may promote self-aggregation or polymer formation, thereby reducing aqueous solubility, receptor-binding affinity, and overall biological activity [54]. In addition, although longer fatty acid chains enhance albumin affinity and prolong circulation time, they may impair transmembrane transport because of increased membrane retention, thereby limiting absorption and tissue distribution [55].

Moreover, the effects of lipidation are highly dependent on the modification site, linker length, and fatty acid type. Different modification patterns may alter peptide conformation and affect receptor recognition. Therefore, extensive structural optimization and biological validation are often required during drug development [56]. In addition, under high concentrations or specific physiological conditions, some lipidated peptides may induce membrane toxicity, nonspecific cellular uptake, or immunostimulatory responses, creating potential safety concerns [57]. Consequently, achieving an optimal balance among prolonged circulation half-life, enhanced bioavailability, and preserved biological activity remains a major challenge in the development and application of lipidation-based modification strategies.

#### 2.2.3. Glycosylation Modification

As a chemical modification strategy for peptide drugs, glycosylation has attracted significant attention for its ability to regulate molecular hydrophilicity, solubility, and serum stability. Sugar moieties typically enhance the structural and physicochemical properties of peptides, thereby increasing resistance to proteolytic degradation and prolonging circulation time in vivo. This effect is frequently accompanied by reduced cytotoxicity and enhanced immunocompatibility [58]. Moreover, glycosylation modifications improve receptor-mediated targeted delivery by interacting with specific cell-surface receptors. For instance, introducing GalNAc ligands significantly increases hepatocyte-specific endocytosis via the asialoglycoprotein receptor (ASGPR), thus enhancing tissue specificity and liver-targeting efficacy [59]. Current studies show that glycosylation of antimicrobial peptides can maintain antimicrobial effectiveness. It can also improve stability and reduce cytotoxicity toward mammalian cells. These outcomes are partially due to optimized hydrophobic/hydrophilic balance and receptor-mediated intracellular uptake mechanisms [58,60]. Recent research also reports that O-glycosylation modifications can attenuate aggregation tendencies of certain pathogenic peptides by inhibiting their aggregation and reducing endogenous toxicity. This finding indicates that glycosylation offers promising applications in peptide drug optimization and modulation of disease-related peptide toxicity [61].

Despite these advantages, glycosylation still has certain limitations. On one hand, glycosylation enhances interactions between peptide chains and water molecules, thus improving solubility, conformational stability, and reducing nonspecific protein adsorption and immunogenicity. It also enhances tissue-targeted delivery efficiency via receptor recognition, with the GalNAc-ASGPR system being particularly significant for liver-targeted therapies [62,63]. On the other hand, glycosylation may alter the binding affinity between peptides and target receptors due to variations in sugar chain type, attachment sites, and degree of modification, potentially reducing biological activity. Additionally, synthesis and precise modification of complex glycan structures typically involve process complexity, high costs, and difficulties in maintaining batch consistency [64]. Furthermore, the metabolic behavior, glycan heterogeneity, and long-term safety of glycosylated peptide drugs require systematic investigation. The exact mechanisms by which different glycan structures influence pharmacokinetics and tissue distribution remain incompletely understood [64].

Recent findings indicate that glycosylation, such as O-GlcNAc modification, not only improves peptide stability but also reduces the toxicity of pathogenic peptides by regulating molecular conformation and preventing abnormal aggregation. For example, in peptides linked to neurodegenerative diseases like α-synuclein, O-glycosylation significantly inhibits their aggregation and pathogenic spread [65,66]. Thus, future developments in glycosylation will focus on enhancing peptide delivery efficiency and bioavailability, integrating strategies such as precise glycoengineering, receptor-mediated targeting, and controlling pathogenic peptide aggregation. The goal is to achieve synergistic optimization of efficacy, safety, and tissue selectivity.

### 2.3. Co-Modification of Main and Side Chains

As the requirements for peptide drug design increase, a single chemical modification often cannot meet all demands. These demands include stability, pharmacokinetics, membrane permeability, and targeting capability. Therefore, combining multiple chemical modification techniques has become a critical direction for peptide drug optimization. Layering different structural modifications onto a single peptide molecule can create molecular-level synergistic effects, providing superior overall performance compared to single modifications [53].

A typical combinational strategy involves combining cyclization and lipidation, which is widely utilized in the development of long-acting antimicrobial and anticancer peptides. Cyclization primarily enhances conformational stability and resistance to enzymatic degradation, while lipidation promotes albumin binding and improves membrane interactions. The synergy of these modifications significantly prolongs circulation time in vivo and enhances cellular targeting efficiency [52,67].

Additionally, the combination of PEGylation and D-amino acid substitution is frequently employed in therapeutic peptides requiring long-term administration. PEGylation extends half-life by forming a hydrated protective layer, while D-amino acid substitution significantly improves proteolytic resistance by disrupting protease recognition sites [58]. Building upon this foundation, introducing glycosylation further enhances solubility, reduces systemic toxicity, and enables receptor-mediated tissue targeting [59].

However, as the number and complexity of modifications increase, combinational modifications also face new challenges. These include more complex synthesis procedures, increased difficulty in structural characterization, limited available modification sites, and potential problems like steric hindrance or functional interference between different modifications [67]. Furthermore, systematic studies on conformational changes, metabolic pathways, and long-term safety following multiple modifications remain limited, and their pharmacokinetics and tissue distribution require further investigation [44,67].

In conclusion, combinational modification strategies effectively overcome limitations associated with single-chemistry modifications in peptide drug optimization. Through synergistic effects, these strategies comprehensively improve stability, bioactivity, pharmacokinetics, and targeted delivery. This approach has become a key research direction in peptide chemical engineering [53]. Future advances in precise modification technologies, sophisticated drug design platforms, and rapid progress in AI-assisted molecular optimization are expected to further enhance stability, bioavailability, and tissue selectivity, while reducing toxicity. This will broaden prospects for research, development, and clinical translation of next-generation precision therapeutic peptides.

## 3. Synthetic Biology Modification Strategies for Bioactive Peptides

### 3.1. Main-Chain Modification Sequence Engineering

#### 3.1.1. Amino Acid Substitution

D-amino acids, due to their distinct stereochemical configuration compared to natural L-amino acids, significantly reduce protease recognition of peptide chains. It should be noted that the effects of D-amino acid incorporation depend on the extent of substitution. Limited substitution of selected L-amino acids with D-enantiomers can enhance protease resistance while generally preserving the overall peptide conformation and biological function. However, complete replacement of all L-amino acids with D-amino acids results in an inversion of the peptide’s overall chirality while maintaining identical side-chain connectivity, generating a mirror-image peptide conformation. This mirror-image configuration may substantially alter molecular recognition with biological targets and therefore requires careful optimization to balance enhanced stability and retained activity [68,69]. This modification enhances the protease stability of peptide drugs, extends their in vivo duration of action, and improves their membrane interaction to a certain extent. This stereoisomeric substitution explains the molecular basis for enhanced protease tolerance in D-amino acid peptides by weakening enzyme-substrate binding affinity [70]. Additionally, incorporating D-amino acids into certain transmembrane peptides and antimicrobial peptide systems has been reported to enhance membrane affinity and cellular uptake efficiency, thereby improving overall bioavailability [71].

Advances in synthetic chemistry and synthetic biology have facilitated the purposeful incorporation of various non-natural amino acids into peptide sequences. Examples include probe amino acids with fluorescent groups, alkyne or azide residues for click chemistry, and building blocks with specific hydrophobic or charged side chains. These modifications not only improve membrane permeability and peptide stability but also enable imaging, targeted delivery, or theranostic applications [35,71]. The use of non-natural and non-standard amino acids expands the physicochemical and functional properties of peptide therapeutics. It is therefore an important strategy for improving receptor selectivity, enabling complex functionalization, and enhancing pharmacokinetic performance [71].

However, while amino acid substitutions can significantly enhance the metabolic stability, receptor-binding specificity, and membrane permeability of peptide drugs, certain limitations remain. On the one hand, extensive incorporation of D-amino acids or non-natural amino acids may alter the original secondary structure and conformational characteristics, weakening target recognition and reducing biological activity. On the other hand, the synthetic pathways for complex non-natural amino acids are lengthy and costly, increasing difficulties in process scale-up and quality control.

Moreover, the metabolic pathways of non-natural residues in vivo are not yet fully elucidated. The long-term biosafety, immunogenicity, and toxicity of potential metabolites require further systematic evaluation. Recent studies indicate that although non-standard amino acids can confer improved pharmacokinetics and functionality to peptide drugs, their design processes face challenges such as predicting SARs, managing increased structural complexity, and addressing limited scalability. Thus, combining computational design, high-throughput screening, and structural biology analyses is typically necessary to precisely optimize molecular properties, balancing enhanced stability with biological activity preservation [72,73,74].

#### 3.1.2. Truncation and Hybridization (Sequence Deletion)

Sequence truncation offers structural optimization by shortening the peptide chain to its minimal active core area. This significantly reduces synthesis and production costs while often increasing membrane permeability and in vivo stability. By accurately identifying and retaining key active residues while removing redundant sequences, this technique maintains or even enhances the inherent biological activity of the peptide. It serves as a classic design strategy in the optimization of peptide drugs [75].

By combining different functional modules into a single peptide molecule, modular design accomplishes the synergistic integration of several biological functions. For example, integrating a drug-carrier module, a receptor-targeting recognition fragment (RGD), or a cell-penetrating sequence can concurrently give the peptide the ability to target, penetrate cells, and deliver cargo. This approach exhibits broad potential applications in multifunctional peptide synthesis, precision cancer therapy, and drug delivery systems. Its key advantage lies in the integration of multiple modes of action into a single molecular scaffold, thereby enhancing the overall selectivity and therapeutic efficacy [76,77].

At the same time, constructing hybrid or modular peptides can achieve synergistic effects among different active fragments. This approach notably improves targeted delivery efficiency, increases local drug concentrations, and reduces nonspecific toxicity. However, increasing the number of functional modules makes peptide molecules more complex and heavier. This creates synthetic difficulties, increases production costs, and complicates pharmacokinetic prediction. Additionally, spatial steric hindrance or conformational interference may occur between functional modules, affecting their independent functions and overall stability.

Multifunctional hybrid peptides may also undergo more complex metabolic pathways in vivo, raising concerns about immunogenicity risks and unpredictable tissue distribution. Their long-term safety and clinical translation efficiency thus require further systematic validation.

Consequently, the design process must balance structural simplification, functional enhancement, and biosafety. Integrating computational design, AI-driven prediction, and high-throughput screening can facilitate more precise structural optimization and functional regulation, enhancing clinical potential and translational feasibility [78,79,80].

### 3.2. Side-Chain Modification

The goal of side-chain engineering, a highly functional molecular modification technique, is to increase peptide biofunctionality by adding particular functional groups to the side chains of amino acids. One widely used method for side-chain modification is fluorescent probe labeling. The stable conjugation of fluorescent probes enables real-time in vivo tracking of the distribution and absorption processes of peptides [81].

In the side-chain design of metal ion coordination sites, functional groups like thiol or imidazole groups that can form stable complexes with metal ions are introduced. This method not only regulates the three-dimensional conformation and structural stability of peptide molecules but also endows them with catalytic or sensing properties associated with metal complexation. It holds great promise for applications in metal ion monitoring and cellular imaging [82,83].

However, controlled release of drugs or functional molecules can be achieved by introducing degradable linkers via side-chain engineering, such as enzyme-sensitive ester bonds or disulfide bonds. This strategy improves targeting and safety in drug delivery systems [84]. Peptide–drug conjugates (PDCs), a crucial application of side-chain engineering, involve covalently linking drug molecules to peptide side chains via cleavable linkers. This combines drug-specific characteristics with peptide targeting and biocompatibility, enabling intracellular responsive drug release. Such an approach significantly increases therapeutic efficacy and reduces adverse effects, establishing PDCs as integral to precision therapies, notably in cancer treatment [84,85]. Importantly, peptide-based conjugation strategies have also demonstrated successful clinical translation. PDCs, such as Lutathera (^177^Lu-DOTATATE) and Vipivotide tetraxetan (^177^Lu-PSMA-617), have been approved for targeted therapy, highlighting the potential of peptide functionalization to enhance tumor targeting and therapeutic precision. These examples demonstrate that rational peptide engineering and conjugation approaches can effectively bridge molecular design and clinical applications.

Overall, side-chain modification strategies offer advantages such as flexible modification sites, strong functional expansion capabilities, and precise functionalization. These modifications endow peptides with multiple functions, including imaging, target recognition, drug delivery, stimulus-responsive release, and biosensing, without significantly altering the peptide backbone conformation. Consequently, these strategies are widely used to develop multifunctional peptide drugs and integrated diagnostic–therapeutic systems. Additionally, by rationally selecting side-chain modification sites and coupling methods, peptide solubility, circulation stability, and tissue distribution characteristics can be enhanced, improving their pharmacokinetic performance.

However, side-chain engineering has limitations. First, introducing functional groups or bulky ligands may cause steric hindrance, disrupting the specific recognition between peptides and their receptors, thus impairing biological activity. Second, various side-chain modifications may induce conformational changes or charge distribution alterations, influencing peptide folding and membrane interactions. Moreover, complex side-chain coupling typically involves multi-step chemical modifications and purification processes, potentially resulting in lower yields, increased structural heterogeneity, and greater quality control difficulties.

For highly functionalized systems such as PDCs, linker stability and cleavability must be carefully balanced. Premature release may cause off-target toxicity, whereas insufficient release could reduce therapeutic efficacy. Additionally, the in vivo metabolic behaviors of fluorescent groups, metal coordination structures, and exogenous functional molecules remain incompletely understood. Their potential immunogenicity, bioaccumulation risks, and long-term safety require further evaluation.

Future advancements in side-chain engineering must integrate site-specific bioconjugation technologies, smart responsive linker designs, and AI-assisted structural optimization strategies. These approaches aim to minimize structural complexity while maintaining functionalization, enabling the development of more efficient, safer, and clinically translatable peptide drugs [84,85,86,87].

### 3.3. Combining Main-Chain and Side-Chain Modifications

Notably, combining side-chain optimization and sequence engineering is a key tactic in the development of modern peptide drugs, which involves improving structural stability by introducing D-amino acids into the backbone, rearranging the sequence, or optimizing the minimal active motif in conjunction with side-chain functionalization (e.g., degradable linkers, metal coordination sites, or fluorescence labeling). By simultaneously improving peptide bioactivity, membrane permeability, and multifunctional characteristics within a single molecular system, this method makes it possible to create functionalized peptide molecules with integrated therapeutic and diagnostic potential [81,88].

For this integrated design, ribosomally produced and post-translationally modified peptides (RiPPs) serve as natural templates. Using specific post-translational modification enzymes, this approach enables cyclization, cross-linking, glycosylation, or disulfide bond formation through the rational design of precursor peptide sequences. This method yields biologically potent and structurally sophisticated antibacterial or anticancer peptides [89,90]. Recently, CRISPR/Cas technology has been widely used to precisely edit and regulate RiPP biosynthesis pathways, enabling in situ side-chain modification and sequence optimization in transgenic bacteria. This significantly improves structural consistency and yield [91,92]. Building on this foundation, peptides can evolve from single-function entities to multi-domain, multi-purpose structures through additional integration with chemical side-chain engineering, such as PDCs or stimulus-responsive linker designs. These developments further enhance their translational potential in cancer treatment, vaccine development, and anti-infective therapies [20,93,94].

Compared with single-modification strategies, the synergistic optimization of main-chain and side-chain modifications fully leverages their complementary strengths: main-chain engineering primarily enhances peptide conformational stability, resistance to proteolytic degradation, and pharmacokinetic properties; side-chain engineering imparts additional functionalities such as target recognition, stimulus-responsive release, imaging and tracing, and drug delivery. Thus, the combination synergistically improves structural stability and functional diversity.

However, as modifications increase, peptide structural complexity significantly rises. Conformational coupling effects, steric hindrance, or charge distribution changes between different modification modules may affect peptide folding and target-binding capacity. Multifunctional peptides typically require complex chemical synthesis, biomanufacturing, and purification, leading to higher production costs and greater demands on quality control and batch consistency.

Systematic studies of the in vivo metabolic behavior, immunogenicity, tissue distribution, and long-term safety of highly functionalized peptides remain limited. Consequently, the clinical translation efficiency of some drug candidates is somewhat constrained. Moreover, predicting synergistic or antagonistic effects among numerous modifications remains challenging using traditional empirical methods. Therefore, employing AI-assisted design, structural biology analysis, high-throughput screening, and synthetic biology platforms is crucial for systematically optimizing the synergy between main-chain and side-chain modifications. This approach facilitates a dynamic balance and precise regulation among multiple parameters.

In the future, integrating advanced techniques such as RiPP engineering, CRISPR-based precision editing, genetic code expansion with non-natural amino acids, and smart responsive linker designs will enhance multifunctional peptide drugs toward greater efficacy, precision, and personalization. These advancements will broaden applications in cancer therapy, anti-infective treatments, and precision medicine [67,89,95].

**Figure 2:**
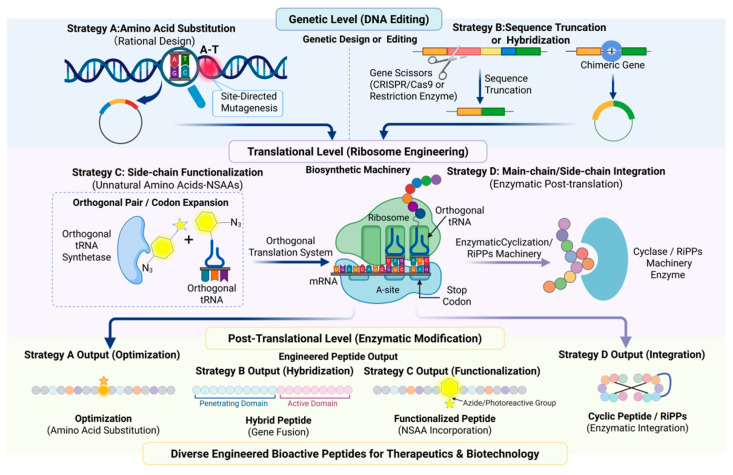
Schematic illustration of bioactive peptide modification based on synthetic biology. Created in BioRender. Hu, L. (2026) https://BioRender.com/klg0iwy [96].

In summary, main-chain sequence reconfiguration, side-chain functionalization, and synergistic optimization of main and side chains represent three synthetic biology-based modification techniques. Figure 2 illustrates the general architecture enabling efficient and logical bioactive peptide creation.

### 3.4. Comparison and Analysis of Various Modification Strategies

As bioactive peptide research advances, single modification strategies are no longer sufficient to simultaneously address multiple requirements such as stability, bioavailability, targeting, and functional diversity. Different modification and engineering methods possess distinct characteristics regarding mechanisms, application scope, and efficacy. Chemical modification primarily improves peptide pharmacokinetic properties and in vivo stability through structural adjustments. In contrast, synthetic biology-based engineering focuses on precise sequence-level regulation to optimize structure and function. Recently, synergistic applications of multiple strategies have become a major research focus. These approaches aim to integrate the advantages of different methods and comprehensively optimize peptide drug performance. To illustrate how different modification strategies have been applied in practice, Table 1 summarizes representative examples of bioactive peptides modified by chemical or synthetic biology-based approaches. In addition to listing representative cases, the table provides a comparative overview of the primary mechanisms, evidence levels, key advantages, and major limitations of each strategy, thereby highlighting their relative strengths and constraints in terms of stability enhancement, bioavailability improvement, manufacturing feasibility, and translational potential.

Table 1 is intended to present representative examples rather than an exhaustive list of all modified bioactive peptides. These examples were selected to illustrate how different modification strategies address specific limitations of native peptides, including poor stability, short half-life, limited membrane permeability, and insufficient bioavailability. As shown in Table 1, different modification strategies exhibit distinct functional emphases. Terminal modifications, cyclization, and backbone modifications primarily improve the pharmacokinetic properties of peptide drugs by enhancing structural stability and resistance to enzymatic degradation. In contrast, conjugation-based or side-chain-related modifications, including PEGylation, lipidation, and glycosylation, are mainly used to prolong circulation time, improve delivery efficiency, and optimize tissue targeting. Synthetic biology-based strategies, such as D-amino acid substitution, incorporation of non-natural amino acids, and modular design, enable functional reprogramming and performance enhancement at the sequence level.

However, these representative cases also indicate that each strategy has inherent trade-offs. Strategies that strongly improve stability or bioavailability, such as cyclization, backbone modification, PEGylation, lipidation, and combined modification, often involve increased synthetic complexity, more demanding purification and characterization procedures, or potential safety concerns. By contrast, simpler approaches such as terminal modification, sequence truncation, and selected amino acid substitution are generally more feasible for synthesis and optimization, but their effects on half-life extension, targeting, or functional diversification may be more limited. Therefore, the selection of an appropriate modification strategy should consider not only biological activity, but also stability, bioavailability, manufacturability, safety, and translational potential.

To further provide practical clinical context, representative FDA-approved modified peptide or peptide-related drugs are summarized in Table 2. These examples illustrate that structural optimization of peptides has contributed to the successful development of clinically used therapeutics.

In recent years, the combined optimization of backbone and side-chain modifications has emerged as a major direction in peptide engineering. By integrating multiple modification strategies, simultaneous improvements in stability, bioactivity, targeting capability, and theranostic functions can be achieved. This approach provides a promising framework for the development of next-generation high-performance multifunctional peptide therapeutics.

## 4. Challenges and Prospects

### 4.1. Challenges Faced

Currently, insufficient site specificity and structural consistency represent two major challenges in the field of peptide engineering. Both in solid-phase chemical synthesis and biological expression processes, precise regulation of amino acid insertion sites and post-translational modifications remains a formidable challenge. Increased product heterogeneity frequently results from this, which affects biological activity, batch consistency, and clinical dependability. In order to improve the structural controllability of peptide molecules, it is commonly acknowledged that creating enzyme-catalyzed site-specific modification techniques and further refining synthetic methodologies are essential [109,110]. Furthermore, a major obstacle in the translation of peptide drugs is insufficient predictive capacity for in vivo behavior. Complicated in vivo physiological conditions, including dynamic pH changes, various protease profiles, and the combined impacts of the immune system, are difficult for traditional in vitro models to accurately duplicate. Predicted and observed in vivo stability and bioavailability frequently differ as a result. To improve the predicted accuracy of the in vivo efficacy of peptide drugs, it is imperative to integrate computational simulations, advanced animal models, and systematic pharmacokinetic studies [111,112]. Meanwhile, evaluations of potential toxicity and immunogenicity remain inadequate. Most current research relies on in vitro models and lacks comprehensive, long-term in vivo data support. Therefore, the development of standardized, high-throughput, and more physiologically relevant safety screening tools is regarded as a critical step in advancing peptide drugs from the laboratory to clinical applications [113,114].

Peptide stability in clinically relevant environments is another important factor affecting the translation of modified bioactive peptides. In dental and oral applications, peptides may be exposed to saliva, salivary enzymes, host- and bacteria-derived proteases, and fluctuating pH conditions, all of which can accelerate peptide degradation or alter peptide conformation. Similarly, peptides intended for gastrointestinal or systemic medical applications may encounter acidic gastric conditions, digestive enzymes, serum proteases, inflammatory exudates, or wound-associated proteolytic environments. These conditions may reduce local retention, shorten biological activity, and limit therapeutic efficacy. Therefore, stability evaluation should not be restricted to simple buffer systems, but should also include physiologically relevant media such as artificial saliva, simulated gastric fluid, serum/plasma, protease-containing solutions, and disease-related microenvironment models. Modification strategies such as N-terminal acetylation, C-terminal amidation, cyclization, D-amino acid substitution, backbone modification, PEGylation, glycosylation, and nanocarrier-based delivery can improve resistance to enzymatic degradation, pH-induced instability, and rapid clearance. Future studies should incorporate these environment-specific stability assays to better predict the performance of modified peptides in dental and medical applications.

Beyond molecular optimization and preclinical evaluation, regulatory considerations represent another important challenge for the clinical translation of modified bioactive peptides. Although chemical and synthetic biology-based modifications can significantly improve peptide stability, bioavailability, and functional properties, these engineered peptides require comprehensive assessment before clinical application, including pharmacokinetic profiles, immunogenicity, toxicity, manufacturing consistency, and quality control. Regulatory agencies such as the FDA and EMA emphasize the necessity of demonstrating both safety and efficacy for modified peptide therapeutics. Several clinically approved modified peptide drugs have demonstrated the feasibility of rational peptide engineering, indicating that appropriate structural optimization can successfully overcome the limitations of natural peptides. However, differences in modification types, production processes, and biological behaviors among engineered peptides make standardized evaluation criteria and predictive models essential for future clinical translation.

### 4.2. Future Trends

In the future, peptide molecular design powered by artificial intelligence (AI) is expected to emerge as a major area of advancement in peptide engineering. It is possible to anticipate amino acid substitutions, conformational stability, and functional–structural connections quickly by employing machine learning and deep learning algorithms to analyze large amounts of sequence and structural data. This speeds up the search for new functional peptides and drastically lowers trial-and-error expenses [115,116]. Recently, several machine learning-based computational frameworks and AI-assisted peptide design platforms have also been developed to facilitate sequence optimization and activity prediction. In peptide engineering, green and sustainable synthesis principles are becoming more and more popular at the same time. These methods focus on microbial fermentation, enzyme catalysis, and renewable feedstocks to reduce the use of organic solvents and the release of chemical waste. This improves yield and purity while also being more environmentally friendly [117,118]. Multimodal delivery methods combine nanocarriers, liposomes, or stimulus-responsive materials at the distribution and application level to accomplish targeted delivery and controlled release of peptide medicines, showing notable benefits in the treatment of chronic illnesses and cancer [119,120]. The design of customized multifunctional peptides has promise for integrating diagnosis, therapy, and efficacy monitoring by further integrating genomic information and patient-specific data. This will propel the evolution of peptide therapeutics from generic therapeutic molecules to precision medicine tools [64,121].

Furthermore, the integration of AI-driven prediction, synthetic biology-based peptide construction, and high-throughput screening platforms will enable rapid identification and optimization of modified bioactive peptides by establishing efficient design–synthesis–evaluation workflows. Recent advances in deep learning, including transformer-based models, graph neural networks, and generative approaches, have further improved peptide activity, stability, and structure prediction from sequence information. These AI-assisted strategies facilitate de novo peptide design and reduce experimental screening costs, accelerating the development of next-generation bioactive peptides [122,123].

## 5. Summary

Bioactive peptides (BPs) represent a multifunctional class of biomolecules with prominent developmental prospects for immunomodulation, antimicrobial therapy, antioxidation and antihypertensive pharmacology. Nevertheless, intrinsic drawbacks including poor in vivo bioavailability and vulnerability to proteolytic degradation necessitate rational structural modification of native peptides.

This review systematically elaborates prevailing chemical modification methodologies, encompassing conjugate derivatization, side-chain functional remodeling and polypeptide backbone engineering. Such chemical modifications prolong peptide half-life and elevate biological potency via steric protection against proteolytic cleavage, strengthened plasma protein binding and improved biomembrane penetrability. In parallel, synthetic biology-based engineering tactics, such as D-amino acid replacement, rational sequence redesign, side-chain derivatization and biosynthetic construction relying on RiPP biosynthetic routes, can be synergized with CRISPR/Cas genome-editing tools to achieve customized molecular design and high-yield biosynthesis of multi-functional bioactive peptides.

Although several bottlenecks remain unaddressed, ranging from unsatisfactory modification site selectivity and unpredictable bioactivity forecasting to incomplete biosafety evaluation, the prospective integration of artificial intelligence-aided peptide design, eco-friendly green synthesis and customized targeted delivery platforms will facilitate the clinical and industrial translational transformation of bioactive peptides, further propelling technological advances in precision medicine and fortified functional food industries.

## Figures and Tables

**Table 1:** Comparison of Major Modification and Engineering Strategies for Bioactive Peptides.

Number	Peptide Name	Modification Strategy	Level of Evidence	Primary Mechanism of Action	Key Advantages	Key Limitations
1	Thymosin α1 [97]	N-terminal acetylation	Clinical	Modulates immune responses by promoting T-cell differentiation and regulating dendritic-cell/macrophage activity	Terminal protection and preserved active peptide structure	Short half-life; repeated dosing required
2	Zilucoplan [98]	Cyclization	Clinical	Inhibits complement C5 cleavage and C5b–C6 binding to block terminal complement activation	Convenient subcutaneous administration; strong complement inhibition.	Risk of meningococcal infection; requires vaccination and safety monitoring
3	Thrombin-binding cyclic peptide [99]	N-methylation	In vivo/preclinical	Promoting the formation of intramolecular hydrogen bonds	Significantly improved oral absorption	Potential reduction in receptor-binding activity
4	Ultravariegin [100]	PEGylation	In vivo/preclinical	Performing spatial shielding and molecular capacitance modification	Extended circulation time in vivo	Reduced activity due to excessive PEG
5	Antimicrobial peptides CGA-N9 [101]	Lipidation	In vivo/preclinical	Enhancing membrane-hydrophobic interactions	Significantly enhanced antifungal activity	Reduced solubility and increased toxicity risk
6	Glycosylated LL-III [60]	Glycosylation	In vitro/ex vivo	Introducing glycosyl steric hindrance at the N-terminal Asn site	Significantly improved protease resistance	Glycosylation sites are residue-specific
7	Teduglutide [102]	Amino acid substitution	Clinical	An Ala-to-Gly substitution at position 2	Improved proteolytic stability and extended half-life	Daily injection; long-term intestinal safety monitoring
8	β-peptide [103]	Introduction of non-natural amino acids	In vitro	Introducing a β-amino acid backbone	Improved structural stability and resistance to proteolytic degradation	The type and arrangement of β-amino acids significantly affect activity
9	Abaloparatide [104]	Sequence truncation and analog design	Clinical	Preservation of the active core	Enhanced bactericidal efficacy	Structural shortening may affect stability and selectivity
10	Cecropin-LL37 Hybrid peptide [105]	Modular hybridization	In vivo/preclinical	Activates PTH1R signaling to promote bone formation	Retains the bioactive PTHrP(1–34) region; broadens the table beyond antimicrobial peptides	Daily injection; duration and skeletal safety require monitoring
11	TMR-RK8 [106]	Fluorescent labeling	In vitro/ex vivo	Embedding imaging functional modules into the peptide chain	No significant reduction in biological activity; enhanced real-time imaging	May slightly affect local spatial conformation, thereby affecting activity
12	Histidine-rich peptide [107]	Metal coordination sites	In vitro	Peptide conformation regulation and functional modular expansion	Conferred multiple biological activities	Insufficient selectivity and potential oxidative toxicity
13	Pleurocidin truncated derivative peptide [108]	Combinations of multiple modifications	In vitro	C-terminal truncation and Trp/Lys substitution synergistically enhance performance	Excellent resistance to pepsin hydrolysis; significantly improved antimicrobial activity	Limited by ion sensitivity and potential membrane toxicity

**Table 2:** Representative FDA-approved modified peptide/protein drugs and their clinical applications.

Number	Generic or Proper Name	Trade Name	Modification Strategy	Therapeutic Indication	Approval Year
1	Enfuvirtide	Fuzeon	Terminal modification: a linear 36-amino acid synthetic peptide with the N-terminus acetylated and the C-terminus a carboxamide	HIV-1 infection in treatment-experienced patients, in combination with other antiretroviral agents	2003
2	Octreotide Acetate	Sandostatin Lar	Cyclization strategy: cyclic somatostatin octapeptide analog containing a disulfide-constrained ring and D-amino acid residue	Reduction in growth hormone; acromegaly; severe diarrhea/flushing associated with carcinoid tumors and VIPomas	1998
3	Pegcetacoplan	Empaveli	Macromolecular polymer modification: two identical cyclic pentadecapeptides covalently linked to a linear PEG molecule	A complement inhibitor indicated for the treatment of adult patients with paroxysmal nocturnal hemoglobinuria (PNH)	2003
4	Semaglutide	Ozempic	Lipidation combined with amino acid substitution: GLP-1 analog containing amino acid substitutions and a C18 fatty diacid side chain for albumin binding	Type 2 diabetes mellitus; chronic weight management	2017
5	Dalbavancin	Dalvance	Glycosylation modification: Semisynthetic lipoglycopeptide with a glycopeptide scaffold and lipophilic side-chain modification	Acute bacterial skin and skin structure infections	2014
6	Degarelix	Firmago	Amino acid substitution: a synthetic linear decapeptide amide containing multiple D-amino acid and non-natural amino acid residues	A GnRH receptor antagonist indicated for treatment of patients with advanced prostate cancer	2008
7	Abaloparatide	Tymlos	Sequence truncation and analog design: a synthetic human parathyroid hormone-related peptide	the treatment of postmenopausal women with osteoporosis at high risk for fracture	2017
8	Lutetium Lu 177 dotatate	Lutathera	Side-chain functionalization (peptide–drug conjugate): DOTA-chelated and radiolabeled somatostatin analog peptide	A radiolabeled somatostatin analog indicated for the treatment of somatostatin receptor-positive gastroenteropancreatic neuroendocrine tumors (GEP-NETs)	2018
9	Tirzepatide	Mounjaro	Co-modification of main and side chains: GIP/GLP-1 receptor agonist peptide with non-natural amino acids and C20 fatty diacid side chain	An adjunct to diet and exercise to improve glycemic control in adults with type 2 diabetes mellitus	2022

Note: This table lists representative FDA-approved examples rather than an exhaustive inventory of all approved peptide drugs. Some drugs involve more than one structural modification and are presented as clinically relevant examples of peptide engineering.

## Data Availability

No new data were created in this review; sharing research data is not applicable.
